# THE EFFECTS OF PARTNERS’ HAPPINESS ON EACH OTHER: AN ACTOR-PARTNER INTERDEPENDENCE MODEL

**DOI:** 10.1093/geroni/igad104.0562

**Published:** 2023-12-21

**Authors:** Elnaz Abaei, Peter Martin

**Affiliations:** Iowa State University, Ames, Iowa, United States; Iowa State University, Ames, Iowa, United States

## Abstract

The study of happiness among older adults has attracted the attention of researchers and practitioners. Happiness is affected by multiple factors, including the relationship with a partner, as they spend more time together in later life. This research aims to evaluate an actor-partner interdependence model, assessing the association of respondent and spouse happiness affecting each other over time. A second aim is to examine the effect of age, gender, education, and whether parents are alive on the respondents’ and their spouses’ happiness. About 9,570 individuals over 50 years of age from the Health and Retirement Study (HRS) and their spouses were included. Respondents’ and their spouses’ happiness measured at two time points were included in the analysis, as were age, gender, education, and the parents’ living status. Multiple regression analyses in SPSS were computed, and the results demonstrated that past happiness is directly associated with present happiness for both respondents and spouses. Respondents’ happiness was also significantly associated with spouses’ happiness, as spouses’ happiness was associated with respondents’ happiness. Furthermore, older age, male gender, and higher education were associated with happiness. Parents’ living status was significantly related only to respondents’ happiness at the first time point, but not to spouses’ happiness. This study helps to understand an actor-partner interdependence model, which highlights partners’ influences on each other’s subjective well-being. Our exploratory study raises several opportunities for future research regarding the actor-partner interdependence model. More research will be necessary to find further variables affecting happiness among older adults.

